# Helper Innate Lymphoid Cells in Allogenic Hematopoietic Stem Cell Transplantation and Graft Versus Host Disease

**DOI:** 10.3389/fimmu.2020.582098

**Published:** 2020-09-30

**Authors:** Linda Quatrini, Nicola Tumino, Francesca Moretta, Francesca Besi, Paola Vacca, Lorenzo Moretta

**Affiliations:** ^1^ Department of Immunology, IRCCS Bambino Gesù Children’s Hospital, Rome, Italy; ^2^ Department of Laboratory Medicine, IRCCS Sacro Cuore Don Calabria Hospital, Negrar, Italy

**Keywords:** innate lymphoid cells, hematopoietic stem cell transplantation, graft *vs* host disease, hematological malignancies, innate lymphoid cell development

## Abstract

Helper Innate Lymphoid Cells (hILCs), including ILC1s, ILC2s, and ILC3s, are mainly localized at the mucosal barriers where they play an important role in tissue regeneration and homeostasis through the secretion of specific sets of cytokines. The recent identification of a circulating ILC precursor able to generate all ILC mature subsets in physiological conditions, suggests that “ILC-poiesis” may be important in the context of hematopoietic stem cell transplantation (HSCT). Indeed, in HSCT the conditioning regimen (chemotherapy and radiotherapy) and Graft *vs* Host Disease (GvHD) may cause severe damages to mucosal tissues. Therefore, it is conceivable that rapid reconstitution of the hILC compartment may be beneficial in HSCT, by promoting mucosal tissue repair/regeneration and providing protection from opportunistic infections. In this review, we will summarize the evidence for a role of hILCs in allogenic HSCT for the treatment of hematological malignancies in all its steps, from the preparative regimen to the immune reconstitution in the recipient. The protective properties of hILCs at the mucosal barrier interfaces make them an attractive target to exploit in future cellular therapies aimed at improving allogenic HSCT outcome.

## Helper Innate Lymphoid Cells

**Figure 1:**
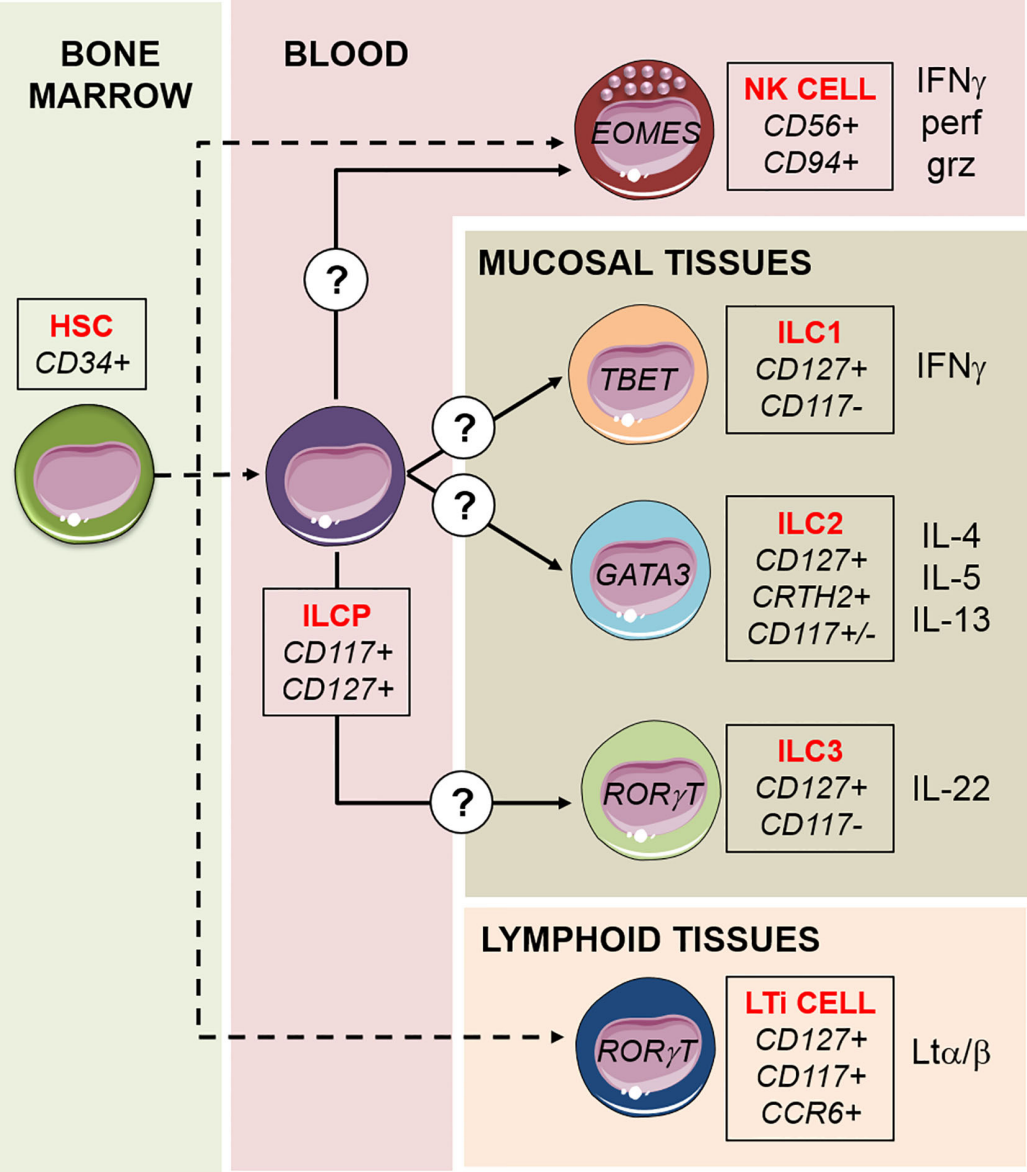
Schematic model of ILCs development. ILCs differentiation proceeds by steps and begins with the hematopoietic stem cell (HSC) in the bone marrow, via intermediate lymphoid restricted precursors (dashed lines). A common ILC precursor (ILCP) circulating in the peripheral blood harbors multipotent capacity to generate NK cells, ILC1s, ILC2, and ILC3s, in response to not completely understood signals. LTi cells are a fifth subset of ILCs, they have a different developmental path compared to the other ILCs and have a crucial role during embryonic development for the formation of lymphoid structures. For each subset, transcription factors, surface markers, and cytokines released are indicated. NK cells are the only “cytotoxic” ILCs, capable of killing target cells through the exocytosis of lytic granules containing perforin (perf) and granzyme (grz).

Innate lymphoid cells (ILCs) comprise five subsets—Natural Killer (NK) cells, ILC1s, ILC2s, ILC3s, and lymphoid tissue inducer (LTi) cells—that represent the innate counterparts of T lymphocytes, as they lack the expression of rearranged antigen-specific receptors (1). In particular, NK cells mirror the functions of CD8^+^ cytotoxic T cells, and the other subsets (ILC1s, ILC2s, and ILC3s, collectively referred to as “helper ILCs”) mirror CD4^+^ T helper (Th)1, Th2, and Th17 cells, respectively, in terms of function (1). While NK cells are mainly circulating in the peripheral blood (PB), helper ILCs (hILCs) are mainly resident at mucosal barrier interfaces, where they play a pivotal homeostatic and protective role. They are activated by inflammatory cytokines and, because they are localized in the lungs, skin, and intestine, their function has mainly been studied in the context of bacteria, parasite, and virus infections at these mucosal sites. ILC1s have several features in common with NK cells: they both produce IFN-γ as their principal cytokine output and require the transcription factor T-bet for this function. In addition, NK cells require Eomes, whereas ILC1s can develop in the absence of this transcription factor, which is, therefore, often used as a marker to distinguish ILC1s from NK cells. In addition, ILC1s are identified by the expression of the surface marker CD127 (shared by all hILC subsets) and the lack of expression of CD117 and CRTH2 (**Figure 1**). ILC1s are generally non-cytotoxic and act as a first line of defense against viruses like murine cytomegalovirus (MCMV) (2), enteric bacteria such as *C. difficile* (3), and parasites like *T. gondii* (4). ILC2s are defined by the expression of higher amounts of the transcription factor GATA3 compared to the other subsets, by the surface expression of CD127 and CRTH2, and by their capacity to produce the type 2 cytokines IL-4, IL-5, and IL-13 in response to IL-25, TSLP, and IL-33 (5, 6) (**Figure 1**). ILC2s are mainly involved in the innate immune response to parasites in the lung and intestine. After resolving the infection, ILC2s contribute to tissue repair by producing amphiregulin (7, 8). ILC3s are abundant at gastro-intestinal (GI) mucosal sites and are involved in the innate immune response to extracellular bacteria and the containment of intestinal commensals (9, 10). ILC3s express CD127 and CD117, produce IL-22 as the predominant homeostatic cytokine, and they are strictly dependent on the transcription factor RORγt (11) (**Figure 1**). ILC3s can be further divided in two subsets on the basis of the cell surface expression of the Natural Cytotoxicity Receptor (NCR), NKp46 in mice and NKp44 in humans (1). Like ILC3s, also a fifth ILC subset, namely LTi cells, is dependent on RORγt and was initially considered to belong to the “group 3” of ILCs. However, LTi cells have a different developmental path compared to other ILCs, and have a crucial role during embryonic development for the formation of secondary lymph nodes and Peyer’s patches, through the action of lymphotoxin (12) (**Figure 1**). LTi cells express CD117 and CCR6, but not NCRs, and are difficult to separate on the basis of marker expression from the postnatal NCR^−^ ILC3s. Indeed, postnatal NCR^−^ ILC3s residing in lymphoid tissues can mediate the formation of tertiary lymphoid structures, and for this reason are sometimes referred to as “LTi-like” ILC3s (13).

A peculiar property of hILCs is that they display a high degree of plasticity, not only during development but also in their mature compartments (1). The plasticity from one subset to the other requires polarizing signals in the tissue in which conversion occurs, together with the expression of cognate cytokine receptors and key transcription factors. For example, IL-22-producing ILC3s conversion into IFN-γ-producing ILC1-like has been documented *in vivo* in mice gut and in the human setting, *in vitro* (14–16). This conversion requires RORγt downregulation, and T-bet and Notch signaling upregulation (14, 17, 18). Also ILC2s can convert into IFN-γ-producing ILC1s both *in vitro* and *in vivo*, upon induction of T-bet and the IL-12 receptor (19, 20). This well-documented hILC subsets plasticity suggests that under the influence of soluble factors or cellular interactions the identity of each subset may not persist *in vivo* upon adoptive transfer, and implies that hILCs functional capability may change substantially in response to the microenvironment.

## Helper ILCs in Hematological Malignancies

Differently from NK cells, whose anti-tumor function has been extensively studied over the last decades (21), the contribution of hILCs in the immune responses against tumors is less clear. From studies investigating hILCs role in solid cancers it seems that they are protective as they can respond rapidly to cytokine stimulation, but their response must be tightly regulated because excessive inflammation can lead to damage and favor tumorigenesis (22). Similarly, sustained secretion of cytokines that promote tissue repair (such as IL-22) can have pathological consequences during chronic activation, inducing epithelial hyperproliferation. Therefore, hILCs act as a double edged sword, and the inflammatory and anti-inflammatory reparative responses that arise during disease must be tightly balanced to prevent tumor development (23).

Much less is known regarding hILCs contribution to hematological malignancies. The data available come mainly from studies analyzing the abundance and function of hILC subsets in patients. For example, in monoclonal gammopathies of undetermined significance (MGUS, representing the earliest lesions leading to multiple myeloma, MM) an increased proportion of ILC1s in the bone marrow (BM) was observed (24). High expression of Ikzf3 in ILC1s together with the high IFN-γ production by ILC1s isolated from pomalidomide-treated patients, suggest that this is among the earliest cell subsets enriched in the tumor microenvironment during evolution of monoclonal gammopathies, and that ILC1s may be a target for immunomodulatory drugs (24). In acute myeloid leukemia (AML), an analysis of the PB from patients before treatment showed a general reduction of hILCs numbers with an increase in the frequency of ILC1s (25). Conversely, in chronic lymphocytic leukemia (CLL) PB hILCs counts are increased and, in particular, ILC1 subset shows a functional impairment, analogous to what was shown for NK cells (26, 27). Also an involvement of ILC2s in hematologic tumors has been reported. Thus, in acute promyelocytic leukemia (APL), elevated tumor-derived prostaglandin-2 (PGD2) and B7H6 induce increases and hyperactivation of ILC2s through binding to CRTH2 and NKp30, respectively. It has been reported that, by releasing IL-13, ILC2 activate myeloid-derived suppressor cells (MDSC) inducing an immunosuppressive pathway (28). Also in AML patients, ILC2s were found to expand in response to the high levels of PGD2 secreted by mesenchymal stromal cells (29). In particular, in this disease PGD2 was shown to induce ILC2s to release IL-5 that, in turn, acts on regulatory T cells stimulating the production of IL-10, promoting proliferation of normal and malignant hematopoietic stem and progenitor cells (29).

Form these evidences it can be concluded that ILC1 dysregulation, in terms of numbers and function, may be implicated in the pathogenesis and progression of diverse hematological malignancies, and that ILC2 induce a tolerogenic environment that favors tumor progression. However, it must be determined whether the phenotype and function reported for hILCs in these patients account for the disease, or they are a consequence of the malignancy itself.

Because of their role both in anti-tumor response in hematologic malignancies and maintenance of epithelial barrier integrity, hILCs represent an attractive tool to exploit in the treatment of these tumors through allogenic hematopoietic stem cell transplantation (allo-HSCT). Indeed, they may play a protective role in the first phase of treatment, when the preparative regimen that precedes transplantation causes mucosal damages. Helper ILCs may then represent a cellular component of the graft and contribute to the defense from infections while the donor-derived immune system develops. Finally, hILCs may be important in the protection from graft *vs* host disease (GvHD), and participate to the immune response that prevents leukemia relapse. Since hILCs have been discovered very recently, the actual contribution of hILCs in these steps has not been fully elucidated. We will summarize in the following paragraphs the evidence that came out of this recent field of investigation, and highlight some interesting aspects that suggest possible advantages of exploiting hILCs in HSCT.

## Allogenic HSCT to Treat Hematological Malignancies

In allo-HSCT, donor-derived HSCs engraft the BM of the recipient and differentiate into mature immune cells, thus reconstituting the recipient lympho/hemopoiesis compromised by either disease or myeloablative therapy. Allo-HSCT is used primarily for hematologic and lymphoid tumors. In adults, the majority of allo-HSCTs are performed for the treatment of acute leukemias, in particular AML. Other major indications include myelodysplastic syndrome or lymphoma (predominantly non-Hodgkin) and, to a lesser extent, MM, chronic myeloid leukemia (CML), and CLL (30). In pediatric patients, allo-HSCT is used also in non-malignant conditions, such as many genetic diseases, including severe combined immunodeficiency (SCID), the Wiskott–Aldrich syndrome, sickle cell anemia, and thalassemia (31).

**Figure 2:**
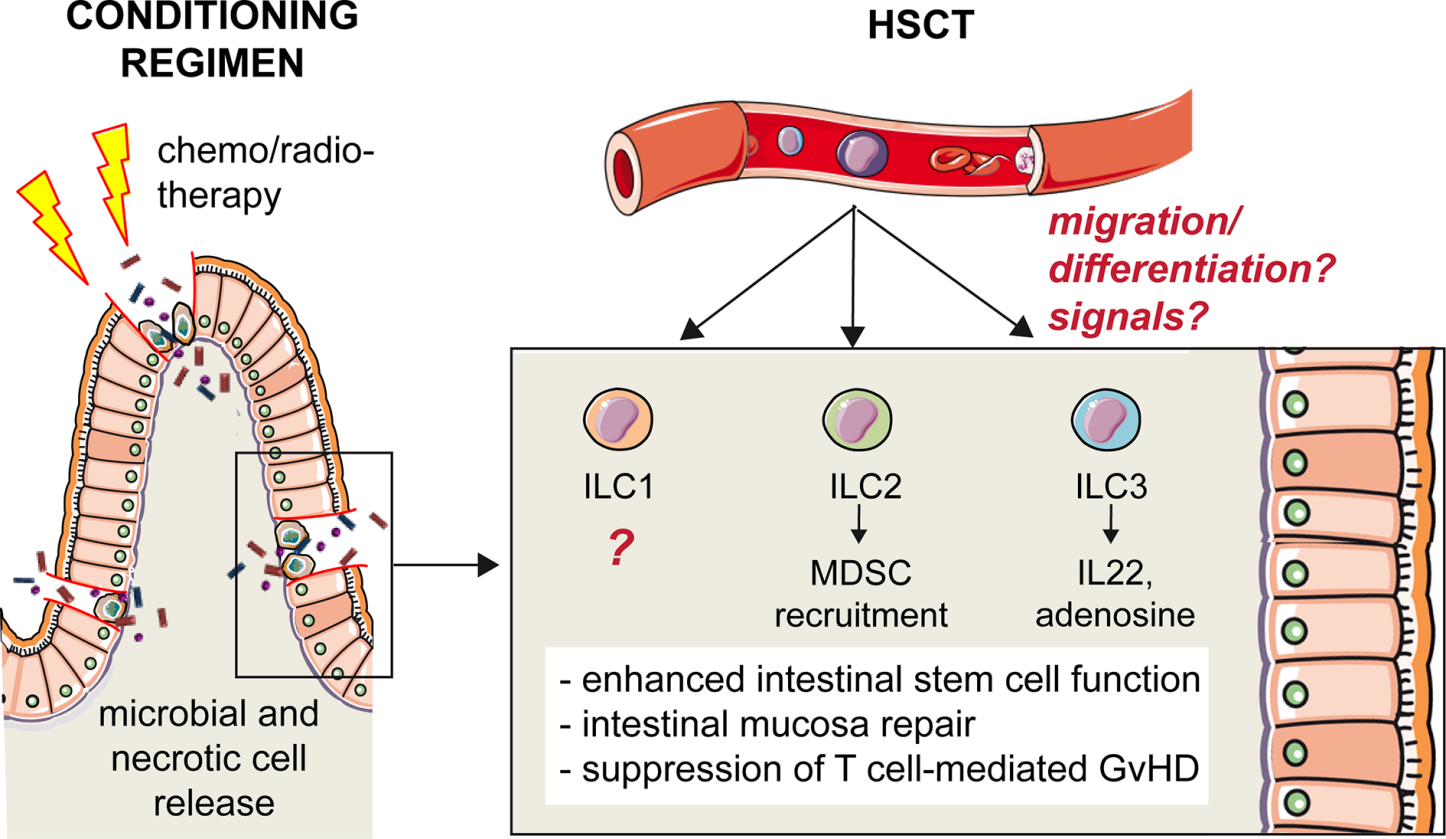
Protective role of hILCs from intestinal GvHD. High-dose preparative regimens that precede HSCT compromise the barrier function of intestinal epithelium and induce the release of microbial and necrotic-cell elements into adjacent tissues and in the bloodstream. The disruption of the intestinal mucosal barrier increases the incidence and severity of GvHD after HSCT. Helper ILCs play a protective role, although it is still not clear what are the signals that trigger these cells and what is their origin. One hypothesis is that a common ILC precursor is recruited from the peripheral blood to the inflamed tissue, and that differentiation occurs locally in response to environmental signals. The role of ILC1s in intestinal GvHD is unknown. ILC2s recruit MDSC that, in turn, suppress T cell-mediated GvHD. ILC3s secrete IL-22 enhancing intestinal stem cell function and promoting repair, and release adenosine, suppressing T cell proliferation.

Prior to transplantation, patients receive conditioning chemotherapy and radiotherapy in order to kill malignant cells and to deplete non-malignant recipient immune cells to avoid rejection (31). Although improving engraftment, preparative regimens cause damages to the mucosae, which favor infections in the mouth, gut, and skin. These damages accentuate and possibly stimulate the occurrence of one of the main complications of allo-HSCT, that is, GvHD (32). In GvHD, the injury is primarily confined to the GI tract, where high dose preparative regimens compromise the barrier function and induce the release of microbial and necrotic-cell elements into adjacent tissues and in the bloodstream (30, 33) (**Figure 2**). A critical role is played by the inflammatory cytokines, such as TNF-α, IL-1, IL-12, and IL-6, that recruit and activate innate immune cells, responsible for antigen presentation and subsequent allo-antigenic response by donor T cells (32). (See also paragraph “*Helper ILCs in graft vs host disease*”).

The predominant source of HSCs for hematologic transplantation is represented by granulocyte colony stimulating factor (G-CSF)-mobilized PB stem cells. In donors who fail to mobilize, G-CSF is administered in combination with plerixafor, a CXCR4 antagonist, inducing an increase of CD34^+^ HSCs in the PB (34). Of note, the *in vitro* development of NK cells and hILCs is influenced by the source of CD34^+^ HSCs and G-CSF treatment (35). Although a major advantage of allogenic grafts is represented by histocompatibility-related immune reactions against tumor cells (Graft *versus* tumor effect), by recognizing recipient antigens, donor T cells also cause GvHD, as mentioned above. Another significant challenge in providing access to HSCT is represented by the availability of a suitable HLA-matched donor. HSCT from a haploidentical donor (haplo-HSCT) offers the option of immediate transplantation virtually to any patients in need of an allograft and lacking suitable HLA-matched donor. To prevent both GvHD and graft failure (36), positive selection of CD34^+^ HSCs and administration of CD34^+^ “megadoses” has been employed for many years (37). However, removal of lymphoid cells and committed hematopoietic progenitors from the graft entails prolonged lymphopenia and delayed immune reconstitution, resulting in an increased risk of non-relapse mortality, mainly from opportunistic infections. A promising approach to circumvent this delay in immune recovery is represented by a method of graft manipulation based on selective depletion of αβ T lymphocytes, responsible for GvHD, and of B cells, from which post-transplant lymphoproliferative disease can arise (38–41). Through this approach it is possible to transfer to the recipient not only donor HSCs but also committed precursors as well as mature NK and γδ T cells, both capable of exerting a protective effect against tumor cell relapse and life threatening infections (42). The role of NK cells in this setting of transplantation has been extensively studied, since it involves an alloreactive mechanism dependent on NK cell expression of inhibitory receptors that interact with class I HLA epitopes (43, 44). The balance between the inhibitory signals from self epitopes and activating signals from ligands expressed by tumor cells improves the chances of engraftment and reduces the risk of GvHD (45). Donor NK alloreactivity can be predicted by analyzing donor KIR phenotype and genotype and HLA class I typing in both donor and recipient, and correlates with a better clinical outcome in both adult AML patients and pediatric ALL and AML patients (42, 46–48). On the other hand, hILCs lack either the expression of KIRs and cytotoxic activity and are therefore unable to exert a direct anti-leukemia effect. Nevertheless, they may indirectly influence the anti-leukemia activity of the other immune cells present in the graft by releasing cytokines. For example, ILC2s may activate a tolerogenic pathway and favor MDSC-dependent suppression of NK function (49), analogously to what was shown in APL (28). Moreover, like NK cells, through cytokine secretion, hILCs may play an important role in the protection against infections during the early time window following HSCT, when the donor-derived immune system is not yet reconstituted.

Although the number of circulating hILC is very low compared, for example, to NK cells, the presence and the function of mature circulating donor-derived hILCs in the graft in HSCT has not been investigated to date. It would be interesting to evaluate in retrospective studies whether there is a correlation between the abundance of infused hILCs in the graft and the clinical outcome of the patient in terms of both relapse and incidence of infections.

More information is available regarding the reconstitution of hILC compartment following HSCT and the role of these cells in GvHD, which are the topics of the following paragraphs.

## Reconstitution of the Helper ILC Compartment

Given the recent discovery of ILCs, our knowledge on their development derives from studies done in the last 10 years. For NK cells, it is well established that development occurs through discrete steps, from stage 1 CD34^+^ NK cell progenitors to stage 4 CD56^bright^ NK cells (50). By *in vitro* experiments using CD34^+^ HSC, it was shown that CD117^high^CD56^+^CD94^-^ stage 3 NK progenitor cells (51) were capable of generating both CD94^+^CD56^+^LFA1^+^ NK cells and CD56^+^CD117^high^LFA1^−^ ILCs, producing IL-8 and IL-22 (52, 53). IL22-producing ILCs were thus recognized as a separate cell lineage but developmentally related to NK cells. The “stage 3” common progenitor cell population was found to depend on the expression of the transcription factor RORγt (54, 55). Subsequently, it was demonstrated that RORγt expression broadly identifies a CD34^+^CD45RA^+^CD117^+^IL-1R1^+^ progenitor population exclusively found in secondary lymphoid tissues (SLTs: tonsils, lymph nodes, spleen) capable of generating all human ILC subsets, including NK cells (56). More recently, a human ILC precursor circulating in the PB has been identified, which displays properties in common with the multipotent ILC precursor (ILCP) previously found in SLTs. These circulating ILCPs characterized by a CD127^+^CD117^+^ phenotype were previously proposed to represent PB ILC3s (57), but it is now recognized that this cell population is enriched in multipotent ILCPs that can give rise to all hILC subsets as well as NK cells (58). It was shown that the expression of CD56 by this progenitor marks the divergence of a shared NK/ILC3 common developmental pathway from ILC2s (59). In addition, NKp46 was identified as a marker that clearly defines the ILC3-potential, while KLRG1 expression indicates a bias towards ILC2 (60). It was demonstrated *in vivo* that this PB ILCP originates from CD34^+^ HSCP (58), but it remains to be studied what are the intermediate steps in this differentiation trajectory (**Figure 1**). A model of differentiation of ILCP towards ILCs is currently proposed and is defined “ILC-poiesis” (61, 62). According to this model, the presence of circulating CD117^+^ ILCPs that eventually develop into mature ILCs ensures a rapid and localized generation of mature ILCs in the tissues in response to environmental signals. The precise mechanisms remain to be fully clarified, however it is clear that important factors in this process are cytokines that drive the trajectories of differentiation (such as IL-1β, IL-23, IL-12, IL-23, IL-25, IL-33, TSLP), and that maintain activated or dividing cells (IL-2, IL-15, IL-7) (63). Any local inflammation associated to infection or tumor transformation would trigger the cellular sources of these cytokines that is, stromal cells, epithelial cells, and other innate immune cells. How this localized ILC differentiation occurs in physiological condition, and how reconstitution of tissue resident ILC compartment occurs in different tissue environments is unclear, but it is likely to recapitulate what happens in infection and inflammation.

Very few studies investigated ILC reconstitution after HSCT. Vely et al. in 2016 studied a cohort of adult patients with SCID who underwent HSCT (64). They found that SCID patients were ILC deficient, and continued to display ILC deficiency after HSCT in the absence of a conditioning regimen to induce myeloablation, possibly because of competition with endogenous progenitors in the appropriate niches. Interestingly, the complete lack of ILCs was not associated to higher susceptibility to diseases, suggesting that, in the conditions of modern medical care and hygiene and in the presence of a functional adaptive immune system, ILCs may be redundant (64). Upon myeloablation, circulating and tissue resident ILCs of donor origin were detected. NK cell differentiation from HSCs requires 2–3 weeks to reach the maturation stage of NKG2A^+^KIR^−^ cells, and the first appearance of KIR^+^, cytolytic, and potentially alloreactive NK cells requires 4–6 additional weeks (45). Conversely, ILC reconstitution is much slower and is incomplete 6 months after allo-HSCT (65). Vely et al. propose that, analogously to tissue-resident macrophages that originate both from yolk sack progenitors and BM HSCs (66), ILCs may originate from a dual pathway: precursors that seed in tissues (probably SLT) during embryonic life, responsible for self-renewal, and BM after birth (64). It would be interesting to verify this hypothesis, to promote hILC reconstitution in tissues from BM precursors and improve protection from GvHD in HSCT.

## Helper ILCs in Graft *vs* Host Disease

Because of the clinical manifestations of GvHD at the mucosal barriers and in particular in the gut, a role for ILC3s in GvHD immunity was hypothesized. Indeed, the critical involvement of hILCs was evidenced for the first time in a mouse model of acute intestinal GvHD (67). In this allo-HSCT model IL-22 producing ILC3 enhanced intestinal stem cell functions, and IL-22 deficiency resulted in increased incidence and severity of GvHD with excessive epithelial cell apoptosis and disrupted intestinal mucosal barrier (67). The main source of IL-22 were NKp46^-^ ILC3s that, importantly, were of recipient origin, as they persisted following lethal conditioning radiotherapy, BM transplantation and even after T cell reconstitution in the *lamina propria* (67). IL-22-mediated epithelium protection by ILC3s is important also in the thymus upon transplantation and GvHD. Indeed, thymus is extremely sensitive to alloreactive damage, mediated by donor-derived T cells expressing IL-21 receptor (68, 69). ILC3s in the thymus are depleted upon GvHD, and it was shown that, preventing ILC3 loss, thymic regeneration and T cell reconstitution are enhanced (69). Thus, through IL-22 production, ILC3s not only favor epithelial regeneration protecting the recipient from GvHD, but also contribute to the restoration of adaptive immunity, which is a critical determinant of successful outcomes in allogenic HSCT.

Apart from maintaining and repairing epithelial barrier integrity through IL-22 secretion, ILC3s have additional modes of action to protect against GvHD. This is suggested by the identification of a novel subset of human ILC3 in the oral-GI tract and in the BM, co-expressing the ectoenzymes CD39 and CD73 (ecto ILC3s) (70). These cells are immunosuppressive because they release adenosine, suppress T cell proliferation and are depleted in patients with GvHD (70).

Different from ILC3s, it was shown that ILC2s in the GI tract but not in the lungs are highly sensitive to conditioning therapy prior to allo-HSCT in a murine model, and their reconstitution from donor BM is quite limited (71). In addition, in this model, co-transfusion of IL-33- activated ILC2s and T cells led to the prevention of GvHD, through the recruitment of MDSC in the GI tract (71). Of note, intravenously infused donor-derived ILC2 could migrate to the GI tract and reduce GvHD without affecting the beneficial T cell-dependent Graft *vs* Leukemia (71). Although it did not directly concern the transplantation context, another study demonstrated that ILC2s promote the renewal of intestinal stem cells through IL-13 secretion, activating the β-catenin pathway (72). This suggests that, analogously to ILC3s, also ILC2s may contribute to epithelial regeneration in the gut and GvHD prevention.

In humans, it was shown that ILCs are depleted from the blood of adult patients suffering from ALL and AML who undergo conditioning therapy before allo-HSCT (65). Patients with a relatively rapid recovery of ILC numbers after induction chemotherapy, before allo-HSCT, experienced less mucositis and less acute GvHD after allo-HSCT, as compared to patients with slower ILC reconstitution dynamics (65). Importantly, lower GvHD incidence was associated to higher proportions of activated CD69^+^ ILCs, expressing tissue homing markers for gut (α4β7, CCR6) and skin (CCR10 and CLA) (65). Notably, 12 weeks after HSCT, the donor-derived circulating NCR^+^ ILC3 count was higher in patients who did not develop GvHD. These cells may actually represent the CD117^+^ ILCP identified later by Di Santo group (58), suggesting that an expansion of the ILC precursors can eventually be protective from GvHD, thanks to the ability of these cells to migrate to the damaged mucosa in response to inflammatory cytokines and give rise to the specialized ILC subsets. Further studies are needed to understand if this is the case, and to precisely describe the steps in the generation of tissue-resident ILC subsets after HSCT.

## Future Directions

While the role of hILCs in the immune response against hematologic malignancies is still controversial and seems to be dependent on the subset and the type of tumor, it is clear that hILCs are relevant in the protection from GvHD. The newly identified circulating ILCPs may represent an attractive cellular target to exploit to further improve the HSCT clinical outcome, thanks to its ability to provide a rapid substrate for the generation of all ILC subsets in response to specific sets of cytokines. For example, ILCPs generated *in vitro* from HSCs present in the graft might be adoptively transferred to patients receiving HSCT and suffering from GvHD with mucosal tissue lesions to facilitate epithelial regeneration. It would be interesting to verify whether tissue resident hILC reconstitution occurs from circulating ILCPs and study it in parallel with reconstitution in the PB. Indeed, although it has been shown that ILC3s in the thymus and GI tract are resistant to radiation injury (67, 68), it remains to be understood to what extent myeloablative regimens and mucosal damage can lead to tissue-resident hILCs depletion in humans.

Besides increasing the risk of GvHD, mucosal damage induced by conditioning regimens may increase the risk of infections, which occur frequently within the first 3 months after transplantation (73). Therefore, the preservation of hILCs (including ILCPs) in the manipulation of the graft can be envisaged as an efficient strategy to protect the recipient from infections, analogously to what has been done for NK cells and γδ T cells. Moreover, while to date there are no studies comparing the effect of T cell depletion on hILC reconstitution, a positive role for hILCs has been shown in the recovery of the adaptive T cell compartment, through protection of thymic epithelium (69). This protective role may ideally be extended to secondary lymphoid tissues (such as lymph nodes) that are damaged by chemotherapy and irradiation used before allo-HSCT. LTi-like ILC3s identified in these adult lymphoid tissues may contribute to their repair, thus indirectly enhancing the recovery of efficient antigen-specific immune response and reducing the risks of opportunistic infection and relapse. Although it is very difficult to study these LTi-like ILC3s in humans, recent findings in mice showed that embryonic LTi cells are replaced in adult lymphoid tissues by HSC-derived cells (74). Since LTi cells in mice have a specific role in the restoration of spleen integrity after infection (75), it is possible that LTi-like ILC3s deriving from HSCs may contribute to lymphoid tissue regeneration upon transplantation.

In conclusion, the ability of hILCs to rapidly secrete an array of different cytokines in a subset-dependent manner makes them a promising tool to exploit to improve allogenic HSCT outcome, by protecting the recipient from GvHD and infections, and enhancing adaptive immune response reconstitution. The strategies aimed at exploiting the properties of these cells may be: (i) to preserve hILCs present in the graft and infuse them in the recipient; (ii) to generate and expand *in vitro* ILCPs from HSCs in the graft in order to adoptively transfer them in recipient; (iii) to accelerate in the recipient the differentiation of hILC subsets potentially useful for the treatment of severe mucosal damages. To do so, further studies on hILCs differentiation are clearly needed, especially to understand how the signals from the tissue microenvironment “tune” the generation of the appropriate ILC subset from a common HSC-derived precursor.

## Author Contributions

All authors listed have made a substantial, direct, and intellectual contribution to the work and approved it for publication.

## Funding

This work was supported by grants awarded by Associazione Italiana per la Ricerca sul Cancro (AIRC)-Special Program Metastatic disease: the key unmet need in oncology 5X1000 2018 Id. 21147 (LM), AIRC IG 2017 Id. 19920 (LM); RC-2020 OPBG (LM, PV); Ministero della Salute RF-2013, GR-2013-02356568 (PV). LQ has received funding from AIRC and from the European Union’s Horizon 2020 research and innovation program under the Marie Skłodowska-Curie grant agreement no 800924. NT was supported by a AIRC fellowship for Italy.

## Conflict of Interest

The authors declare that the research was conducted in the absence of any commercial or financial relationships that could be construed as a potential conflict of interest.
